# Mapping endocrine toxicity spectrum of immune checkpoint inhibitors: a disproportionality analysis using the WHO adverse drug reaction database, VigiBase

**DOI:** 10.1007/s12020-020-02355-9

**Published:** 2020-06-07

**Authors:** Xuefeng Bai, Xiahong Lin, Kainan Zheng, Xiaoyu Chen, Xiaohong Wu, Yinqiong Huang, Yong Zhuang

**Affiliations:** 1 Department of Endocrinology, The Second Affiliated Hospital of Fujian Medical University, No. 950 Donghai Street, Fengze District, Quanzhou, Fujian Province China; 2 Department of Medical Administration, The Second Affiliated Hospital of Fujian Medical University, No. 950 Donghai Street, Fengze District, Quanzhou, Fujian Province China; 3 Data Mining Working Group, China Telecom Co., Ltd (Quanzhou Branch), No. 105 Citong Road, Fengze District, Quanzhou, Fujian Province China

**Keywords:** Immune checkpoint inhibitor, Endocrine toxicity, Endocrinopathy, Immune-related adverse event, Onset time

## Abstract

**Purpose:**

Our study aimed to map endocrine toxicity spectrum of immune checkpoint inhibitors (ICIs).

**Methods:**

We obtained data from VigiBase, between January 1, 2011 and March 6, 2019. All endocrine adverse drug reactions (ADRs) were classified by group queries according to the Medical Dictionary for Regulatory Activities. Disproportionality analysis was performed with information component (IC) and reporting odds ratio (ROR). We used IC to identify meaningful endocrinopathies associated with ICIs and ROR to compare differences between ICI subgroups of ADRs. IC_025_ (lower end of the 95% confidence interval of IC) is considered significant if larger than 0.

**Results:**

In all, 6089 reports for endocrinopathies associated with ICIs were involved, with a male to female ratio of 1.5:1. The disproportionality analysis indicated significance of not only common endocrinopathies: thyroid dysfunction, hypophysitis/hypopituitarism, adrenal insufficiency, T1DM, fulminant T1DM (IC_025_: 4.12–6.62), but also rare endocrinopathies: hypoparathyroidism, diabetes insipidus, hypogonadism (IC_025_: 1.56–2.04). Increased risk of ADR reporting emerged in anti-CTLA-4 (e.g., hypophysitis/hypopituitarism, adrenal insufficiency) or in anti-PD-1/PD-L1 (e.g., thyroid dysfunction, T1DM, fulminant T1DM). In general, combination therapy (anti-CTLA-4 plus anti-PD-1/PD-L1) had a stronger association with endocrinopathies than monotherapy (ROR: 2.8, 95% CI: 2.5–3.1). Onset time of common endocrinopathies differed between different ICI therapies, typically within 12 weeks in anti-CTLA-4 monotherapy but diffusely ranging from 0 to 48 weeks in anti-PD-1 monotherapy.

**Conclusions:**

Our study shows rising reporting frequencies of endocrinopathies caused by ICIs, especially aggravated in combination therapy. Clinicians should be early aware of latent endocrine toxicity and different onset time of endocrinopathies when implementing ICI therapies.

## Introduction

Immune checkpoint inhibitors (ICIs) have received extensive attentions as one of the most successful immunotherapies to date, which can significantly improve clinical outcomes in multiple cancer types [1]. However, ICIs may also induce a large series of autoimmune adverse events, known as immune-related adverse events (irAE), which are completely different from those of conventional chemotherapy, radiation and other cancer treatments. These irAEs can affect numerous organs in the body, including common organs such as skin, liver, colon, lung, and endocrine system, and less common organs such as kidney, eye, nervous system, cardiovascular system, musculoskeletal system, and hematologic system [2, 3]. Endocrine adverse drug reactions (ADRs) are the most often occurred irAEs with ICI therapies, including thyroid dysfunction (hyperthyroidism and hypothyroidism), hypophysitis, primary adrenal insufficiency, and insulin-deficient diabetes mellitus [4, 5]. Although the severity of most endocrine ADRs is from mild to moderate [6, 7], life-threatening complications may occur if patients do not receive a regular evaluation or treatment [8, 9].

In view of the limitations of randomized clinical trials, often with small sample size and a homogenous population, the analysis of international spontaneous reporting systems allows to map a broader profile by collecting reports of ADRs in real-world submitted worldwide, which ensures us to rapidly detect even rare endocrine ADRs. In this study, we use VigiBase, the WHO global database of individual case safety reports [10], which is managed by the Uppsala Monitoring Centre (UMC) in Sweden, to fully understand the toxicity spectrum and clinical features of ICI-related endocrinopathies.

## Methods

### Study design and data sources

The study was designed as an observational, retrospective, pharmacovigilance study based on VigiBase database. As a spontaneous reporting system, VigiBase allows for disproportionality analysis, by which to detect and quantify the associations between target drugs and suspected ADRs. Reports of ADRs were submitted from multiple sources, such as physicians, pharmacists, other health care professionals, and patients. In VigiBase, all ADRs are coded in line with Adverse Reaction Terminology (WHO-ART) and Medical Dictionary for Regulatory Activities (MedDRA; www.who-umc.org).

### Procedures

**Fig. 1:**
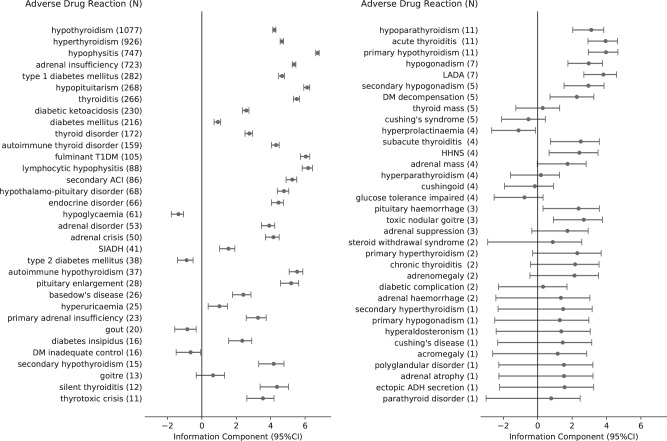
The comprehensive spectrum of endocrine ADRs associated with ICIs reported from VigiBase. Information component (IC) reflects the strength of the drug-adverse event association. Positive IC value is regarded as a significant signal in VigiBase. All endocrine ADRs (*N* = 6089) classified by group queries according to the Medical Dictionary for Regulatory Activities (MedDRA, version 20.1). ICI immune checkpoint inhibitor, ADR adverse drug reaction, SIADH inappropriate antidiuretic hormone secretion, LADA latent autoimmune diabetes in adults, Cushing’s disease pituitary-dependent Cushing’s syndrome, secondary ACI secondary adrenocortical insufficiency. adrenal crisis: acute adrenocortical insufficiency. DM decompensation diabetic metabolic decompensation, DM inadequate control diabetes mellitus inadequate control, HHNS hyperglycaemic hyperosmolar nonketotic syndrome, fulminant T1DM fulminant type 1 diabetes mellitus, ectopic ADH secretion ectopic antidiuretic hormone secretion

Five ICI drugs were analyzed and discussed in this study, including anti-CTLA-4 antibodies (ipilimumab), anti-PD-1 antibodies (nivolumab and pembrolizumab), and anti-PD-L1 antibodies (atezolizumab and durvalumab). Combination therapy refers to the therapy using both ipilimumab and anti-PD-1/PD-L1. The study included all reports of endocrine ADRs associated with ICIs, from January 1, 2011 to March 6, 2019. Each report was coded using Preferred Terms from MedDRA (version 20.1). In this study, the definition of ICI-DM includes the following MedDRA terms: type 1 diabetes mellitus, diabetes mellitus, fulminant type 1 diabetes mellitus, and diabetes ketoacidosis. Other endocrine-related MedDRA terms can be found in Fig. 1.

Endocrine ADRs assessed in the analysis were those submitted as suspected to be caused by ICIs. Before performing statistical analysis, duplicate reports were removed. Each report contained report ID, notifier, country of origin, patient features (gender and age), reason for drug use, dosage regimen, onset time of ADRs, and final outcomes. One patient had a unique report ID, and one report ID may include more than one event. Fatal outcomes were defined as those resulted in death, life-threatening, hospitalization (initial or prolonged), or any other medically critical conditions.

### Statistical analysis

In this pharmacovigilance study, disproportionality analysis was used to compare the proportion of selected ADRs reported for a single drug or a group of drugs (e.g., ICIs) with the proportion of the same ADRs for a control group of drugs. The denominator is the total number of reports of ADRs for each drug or group of drugs. If the proportion of one ADR is significantly greater in patients exposed to a selected drug (cases) than in patients not exposed to this drug (non-cases), then an association can be identified between the selected drug and the ADR. Through this case/non-case analysis, we evaluate whether the occurrence of endocrine ADRs was differentially reported between ICIs (or ICI subgroup) and other drugs.

Reporting odds ratio (ROR) and information component (IC) were used to calculate disproportionality. IC derives from a Bayesian confidence propagation neural network (BCPNN) [11], which reflects the strength of the drug-adverse event association. Unlike ROR, IC can provide a conservative measure of association and reduce the risk of highlighting spurious associations, particularly for ADRs with very low expected frequencies in a large database (e.g., VigiBase) [12]. Therefore, we used IC to identify meaningful endocrine ADRs, while using ROR to compare differences in the reporting of endocrine ADRs caused by different ICI subgroups. Statistical formulas [13] of IC and ROR are as follows:

**Table:** 

	Selected ADR	Other ADRs	Total
Selected drug	A	B	A + B
Contrast drugs	C	D	C + D
Total	A + C	B + D	A + B + C + D

ROR = (A/C)/(B/D)

IC = log_2_ [(A + 0.5)/(*N*_expected_ + 0.5)]

*N*_expected_ = [(A + B) × (A + C)]/(A + B + C + D)

IC_O25_ is defined as the lower bound of 95% confidence interval for IC value and positive IC_025_ is the traditional threshold for statistical significance used in signal detection at the UMC. We implemented a cluster method on all positive IC_025_ and got three clusters (0–3, 3–5, 5+), and therefore we use thresholds of 3 and 5 to divide IC_025_ into three categories (regarded as weak, moderate, and strong association, respectively). On the analysis of ROR, a criterion based on minimum count of drug-adverse event (*N* ≥ 3) was imposed [12]. ROR is identified significant if the lower bound of 95% CI is larger than 1.

Descriptive statistical methods were used to summarize clinical characteristics of endocrine ADRs. The differences of onset time were tested by *t*-test (equaled variance) or Welch’s *t* test (unequaled variances) or ANOVA after a logarithmic transformation. The analysis in our report was performed with Python-3.7.0 and python packages of numpy-1.17.2, scipy-1.1.0, pandas-0.23.4, matplotlib-2.2.2.

## Results

### Descriptive analysis

From January 1, 2011 to March 6, 2019, 13,300,773 ADRs from patients who received any drugs (full database) were reported in VigiBase. In all, 6089 report cases for endocrine ADRs, which contained at least one ICI therapy record, were involved after de-duplication and preprocessing. The demographic data of patients with ICI-related endocrinopathies were described in Table 1. Most cases were observed in patients with malignant melanoma, lung cancer, or renal cell carcinoma. Nivolumab (*N* = 2449, 40.2%) had the largest number of reports, followed by pembrolizumab (*N* = 1342, 22.0%) and ipilimumab (*N* = 1115, 18.3%). Most cases were reported between 2017 and 2019 (>75%), especially for anti-PD-1 monotherapy and combination therapy, reflecting a considerably increasing usage of ICIs in recent years. The reports of endocrine ADRs were mainly from Europe (43.8%), followed by Americas (30.0%), and Asia (23.4%). Among all endocrine ADRs, young adults (<65 years old) and old aged (≥65 years old) had similar proportions, and the male was slightly dominant both in total (male: 60.1% vs. female: 39.9%) and in different ICI subgroups (male: 54.3–68.8%, female: 31.2–45.7%). Health professionals were the main notifiers of all reports (88.4%, peaking at 97.2% for durvalumab), which greatly increased the reliability of the dataset. There were 2483 cases (69.5%, 3572 cases with the final outcomes reported) reported as being in remission or recovered after standard treatment, while death and life-threatening events occurred in 64 (1.8%) cases.

**Table 1:** Clinical characteristics of patients with ICI-related endocrinopathies collected from VigiBase

	ICI (total)	Monotherapy					Combination therapy^d^
		Ipilimumab	Nivolumab	Pembrolizumab	Durvalumab	Atezolizumab	
*N* (%)	6089 (100.0%)	1115 (18.3%)	2449 (40.2%)	1342 (22.0%)	111 (1.8%)	110 (1.8%)	962 (15.8%)
Year							
2011	23 (0.4%)	23 (2.1%)					
2012	80 (1.3%)	79 (7.1%)					1(0.1%)
2013	79 (1.3%)	77 (6.9%)	2 (0.1%)				
2014	174 (2.9%)	166 (14.9%)	1 (0.0%)	1 (0.1%)		1 (0.9%)	5 (0.5%)
2015	407 (6.7%)	233 (20.9%)	39 (1.6%)	77 (5.7%)			58 (6.0%)
2016	676 (11.1%)	159 (14.3%)	316 (12.9%)	92 (6.9%)	1 (0.9%)	6 (5.5%)	102 (10.6%)
2017	1595 (26.2%)	187 (16.8%)	788 (32.2%)	289 (21.5%)	7 (6.3%)	32 (29.1%)	292 (30.4%)
2018	2612 (42.9%)	171 (15.3%)	1100 (44.9%)	730 (54.4%)	83 (74.8%)	64 (58.2%)	464 (48.2%)
2019^e^	443 (7.3%)	20 (1.8%)	203 (8.3%)	153 (11.4%)	20 (18.0%)	7 (6.4%)	40 (4.2%)
Gender							
Female	2279/5711 (39.9%)	395/1030 (38.3%)	879/2314 (38.0%)	515/1241 (41.5%)	34/109 (31.2%)	41/109 (37.6%)	415/908 (45.7%)
Male	3432/5711 (60.1%)	635/1030 (61.7%)	1435/2314 (62.0%)	726/1241 (58.5%)	75/109 (68.8%)	68/109 (62.4%)	493/908 (54.3%)
Age group							
<65	2233/4508 (49.5%)	456/831 (54.9%)	817/1794 (45.5%)	429/1015 (42.3%)	49/84 (58.3%)	32/82 (39.0%)	450/702 (64.1%)
≥65	2275/4508 (50.5%)	375/831 (45.1%)	977/1794 (54.5%)	586/1025 (57.7%)	35/84 (41.7%)	50/82 (61.0%)	252/702 (35.9%)
Region							
Americas	1826 (30.0%)	584 (9.6%)	405 (16.5%)	354 (26.4%)	25 (22.5%)	48 (43.6%)	410 (42.6%)
Asia	1427 (23.4%)	72 (1.2%)	734 (30.0%)	475 (35.4%)	0	8 (7.3%)	138 (14.3%)
Europe	2665 (43.8%)	388 (6.4%)	1289 (52.6%)	469 (34.9%)	82 (73.9%)	52 (47.3%)	385 (40.0%)
Oceania	170 (2.8%)	71 (1.2%)	21 (0.9%)	44 (3.3%)	4 (3.6%)	1 (0.9%)	29 (3.0%)
Africa	1 (0.0%)	0	0	0		1 (0.9%)	0
Notifier^a^							
Notifier1	5220/5906 (88.4%)	782/1024 (76.4%)	2262/2427 (93.2%)	1176/1308 (89.9%)	104/107 (97.2%)	99/109 (90.8%)	797/931 (85.6%)
Notifier2	686/5906 (11.6%)	242/1024 (23.6%)	165/2427 (6.8%)	132/1308 (10.1%)	3/107 (2.8%)	10/109 (9.2%)	134/931 (14.4%)
Indication^b^							
Malignant melanoma	2328/5348 (43.5%)	792/839 (94.4%)	431/2230 (19.3%)	401/1195 (33.6%)	0	2/97 (2.1%)	702/887 (79.1%)
Lung cancer	1912/5348 (35.8%)	2/839 (0.2%)	1189/2230 (53.3%)	532/1195 (44.5%)	83/100 (83.0%)	37/97 (38.1%)	69/887 (7.8%)
Renal cell carcinoma	330/5348 (6.2%)	1/839 (0.1%)	284/2230 (12.7%)	9/1195 (0.8%)	0	7/97 (7.2%)	29/887 (3.3%)
Cancer of head and neck	83/5348 (1.6%)	1/839 (0.1%)	72/2230 (3.2%)	8/1195 (0.7%)	2/100 (2.0%)	0	0
Gastric carcinoma	61/5348 (1.1%)	0	56/2230 (2.5%)	1/1195 (0.1%)	0	0	4/887 (0.5%)
Bronchial carcinoma	49/5348 (0.9%)	0	43/2230 (1.9%)	5/1195 (0.4%)	0	0	1/887 (0.1%)
Urothelial carcinoma	43/5348 (0.8%)	0	6/2230 (0.3%)	11/1195 (0.9%)	0	26/97 (26.8%)	0
Others	542/5348 (10.1%)	43/839 (5.1%)	149/2230 (6.7%)	228/1195 (19.1%)	15/100 (15.0%)	25/97 (25.8%)	82/887 (9.2%)
Final outcome							
Fatal^c^	64/3572 (1.8%)	17/481 (3.5%)	21/1520 (1.4%)	20/862 (2.3%)		2/72 (2.8%)	4/564 (0.7%)
Not recovered/not resolved	1025/3572 (28.7%)	128/481 (26.6%)	423/1520 (27.8%)	281/862 (32.6%)	23/73 (31.5%)	31/72 (43.1%)	139/564 (24.6%)
Recovered/resolved	2483/3572 (69.5%)	336/481 (69.9%)	1076/1520 (70.8%)	561/862 (65.1%)	50/73 (68.5%)	39/72 (54.2%)	421/564 (74.6%)

Data are described as *n* (%), *n*/*N* (%)

^a^Notifier1 refers to physician, pharmacist and other health professional. Notifier2 refers to lawyer, consumer

^b^Indication refers to the primary tumors treated by ICIs, and the complete indications are provided in Attachment 1

^c^A fatal outcome was defined as causing death; being life-threatening; requiring hospitalization (initial or prolonged); or causing any other medically critical diseases

^d^Combination therapies include ipilimumab + nivolumab (*N* = 885), ipilimumab + pembrolizumab (*N* = 50), ipilimumab + nivolumab + pembrolizumab (*N* = 27)

^e^Only contains cases before March 6, 2019

### Disproportionality analysis

#### The comprehensive spectrum of endocrine ADRs

The disproportionality analysis highlighted several ICI-related endocrinopathies with large significant IC values and narrow 95% confidence intervals, mainly invading the thyroid, pituitary, adrenal, and pancreas: thyroid dysfunction, including hypothyroidism (*N* = 1077; IC value: 4.22, 95% CI: 4.12–4.30), hyperthyroidism (*N* = 926; IC value: 4.66, 95% CI: 4.55–4.74), thyroiditis (*N* = 266; IC value: 5.52, 95% CI: 5.32–5.67); pituitary disease, including hypophysitis (*N* = 747; IC value: 6.74, 95% CI: 6.62–6.82), hypopituitarism (*N* = 268; IC value: 6.12, 95% CI: 5.92–6.27); adrenal insufficiency (*N* = 723; IC value: 5.37, 95% CI: 5.25–5.46); pancreas disease, including type 1 diabetes mellitus (*N* = 282; IC value: 4.66, 95% CI: 4.47–4.80), diabetic ketoacidosis (*N* = 230; IC value: 2.58, 95% CI: 2.36–2.74), fulminant type 1 diabetes mellitus (*N* = 105; IC value: 6.05, 95% CI: 5.73–6.27) (Fig. 1). Notably, we also discovered some rare reports with large statistically significance: adrenal crisis (*N* = 50; IC value: 4.16, 95% CI: 3.70–4.49), thyrotoxic crisis (*N* = 11; IC value: 3.55, 95% CI: 2.61–4.20), hypoparathyroidism (*N* = 11; IC value: 3.12, 95% CI: 2.04–3.84), diabetes insipidus (*N* = 16; IC value: 2.35, 95% CI: 1.56–2.90), hypogonadism (*N* = 7; IC value: 2.97, 95% CI: 1.76–3.77).

#### Comparison of endocrine ADRs in different ICI monotherapies

**Fig. 2:**
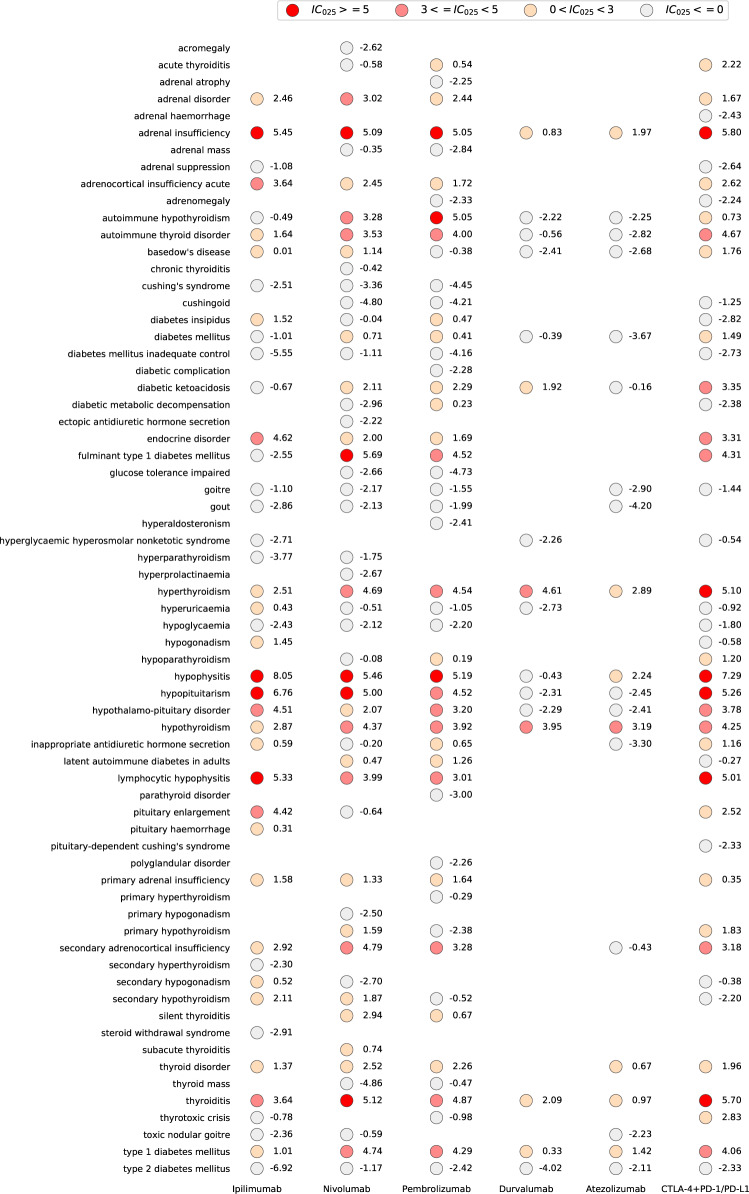
Endocrine toxicity spectra in different ICI therapies. IC information component, IC_025_ the lower end of the 95% confidence interval of IC. IC_025_ > 0 is regarded as statistically significant. Combination therapies include ipilimumab + nivolumab, ipilimumab + pembrolizumab, and ipilimumab + nivolumab + pembrolizumab

With further analysis, different reporting frequencies of endocrine ADRs were observed in different ICI therapies compared with the full database (Fig. 2). For monotherapy, anti-PD-1 treatment had a broader spectrum of endocrine toxicity than anti-CTLA-4 treatment. Anti-CTLA-4 had the strongest signal in hypophysitis (*N* = 394; IC_025_: 8.05). There were other endocrinopathies strongly associated with ipilimumab (IC_025_ ≥ 5.0): hypopituitarism, adrenal insufficiency, lymphocytic hypophysitis, moderately associated with ipilimumab (3.0 ≤ IC_025_ < 5.0): pituitary enlargement, acute adrenocortical insufficiency, thyroiditis, and weakly associated with ipilimumab (0 < IC_025_ < 3.0): hyperthyroidism, hypothyroidism, primary adrenal insufficiency, diabetes insipidus, hypogonadism, type 1 diabetes mellitus, etc. For fulminant type 1 diabetes mellitus, it was observed strongly associated only with anti-PD-1 among all ICI monotherapies (*N* = 64 for nivolumab, IC_025_: 5.69; *N* = 24 for pembrolizumab, IC_025_: 4.52) but unassociated with anti-CTLA-4 (*N* = 1; IC_025_: −2.55) and not reported with anti-PD-L1. Anti-PD-1 monotherapy was also strongly associated with hypophysitis, hypopituitarism and adrenal insufficiency, moderate associated with thyroid dysfunction, type 1 diabetes mellitus, etc. (Fig. 2) Interestingly, we observed Basedow’s disease weak associated with nivolumab (*N* = 11, IC_025_: 1.14).

Anti-PD-L1 induced the narrowest endocrine toxicity spectrum among all ICI monotherapies (Fig. 2), with the least number of reports (*N* = 111 for durvalumab, *N* = 110 for atezolizumab). There were more reports of hyperthyroidism (*N* = 38 for durvalumab, IC_025_: 4.61; *N* = 24 for atezolizumab, IC_025_: 2.89) and hypothyroidism (*N* = 34 for durvalumab, IC_025_: 3.95; *N* = 34 for atezolizumab, IC_025_: 3.19). In addition, anti-PD-L1 also induced some endocrine ADRs reported with statistically weak significance, such as diabetic ketoacidosis, adrenal insufficiency, type 1 diabetes mellitus, and hypophysitis (Fig. 2).

**Fig. 3:**
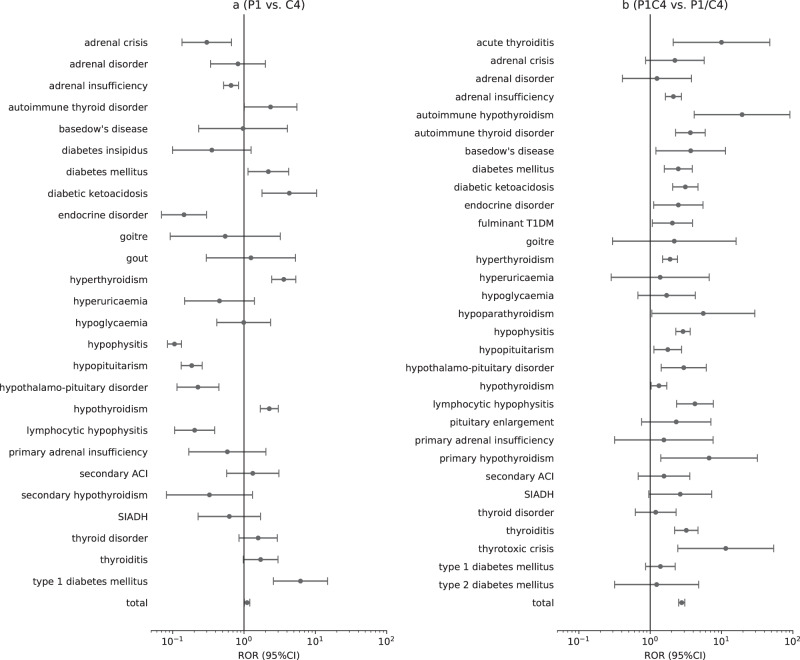
Comparison of endocrine ADRs in different ICI therapies. **a** P1 vs. C4: anti-PD-1/PD-L1 monotherapy vs. anti-CTLA-4 monotherapy. **b** P1C4 vs. P1/C4: anti-PD-1/PD-L1 combined with anti-CTLA-4 vs. anti-PD-1/PD-L1 monotherapy and anti-CTLA-4 monotherapy. On the analysis of ROR, a minimum count of drug-adverse reaction (*N* ≥ 3) was imposed. SIADH inappropriate antidiuretic hormone secretion, secondary ACI secondary adrenocortical insufficiency, adrenal crisis acute adrenocortical insufficiency, fulminant T1DM fulminant type 1 diabetes mellitus

Figure 3 demonstrates the differences in endocrine toxicity spectrum between anti-PD-1/PD-L1 monotherapy and anti-CTLA-4 monotherapy, suggesting that although anti-PD-1/PD-L1 had a larger proportion of reported cases, significant ROR values (higher than 1) were only found in endocrine ADRs related to pancreas and thyroid, such as type 1 diabetes mellitus (ROR 6.2, 95% CI: 2.6–14.8), diabetic ketoacidosis (ROR: 4.3, 95% CI: 1.8–10.4), hyperthyroidism (ROR: 3.6, 95% CI: 2.4–5.3), hypothyroidism (ROR: 2.3, 95% CI: 1.7–3.0).

#### Comparison of endocrine AEs between combination and monotherapy

Combination therapy-related endocrinopathies covered endocrine ADRs induced by anti-CTLA-4 monotherapy and anti-PD-1/PD-L1 monotherapy. Combination therapy was strongly associated with hypophysitis (*N* = 153, IC_025_: 7.29), adrenal insufficiency (*N* = 118, IC_025_: 5.80), thyroiditis (*N* = 57, IC_025_: 5.70), hypopituitarism (*N* = 35, IC_025_: 5.26), and hyperthyroidism (*N* = 131, IC_025_: 5.10). We also observed hypoparathyroidism with a weak association with combination therapy (*N* = 5, IC_025_: 1.20) and a weak association with pembrolizumab (*N* = 3, IC_025_: 0.19), but not reported in ipilimumab. Figure 3 shows that combination therapy had a stronger association with almost all endocrine ADRs than monotherapy and the ROR of total endocrine ADRs was 2.8 (95% CI: 2.5–3.1).

### Onset time of ICI-related endocrinopathies

The onset time of 2765 cases of ICI-related endocrinopathies was reported. We analyzed the characteristics of onset time of some ADRs with cases more than 300 and targeted on four drug subgroups (nivolumab, pembrolizumab, ipilimumab, combination therapy), including hyperthyroidism (*N* = 525), hypothyroidism (*N* = 502), adrenal insufficiency (*N* = 320), hypophysitis/hypopituitarism (*N* = 416). In addition, due to similar characteristics of onset time of these four ADRs: type 1 diabetes mellitus, diabetes mellitus, fulminant type 1 diabetes mellitus, and diabetes ketoacidosis, we grouped them as ICI-DM (*N* = 319) for further analysis.

**Fig. 4:**
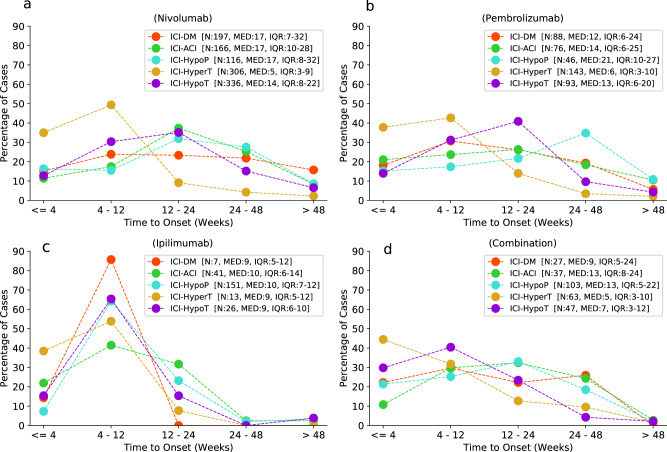
Onset time of major endocrine ADRs associated with **a** (nivolumab), **b** (pembrolizumab), **c** (ipilimumab), **d** (combination). Combination: anti-PD-1/PD-L1 combined with anti-CTLA-4. ICI-ACI ICI-related adrenocortical insufficiency, ICI-HypoP ICI-related hypophysitis/hypopituitarism, ICI-HyperT ICI-related hyperthyroidism, ICI-HypoT ICI-related hypothyroidism

Figure 4 shows that: (1) when using ipilimumab, ICI-DM (*N* = 7), hyperthyroidism (*N* = 13), hypothyroidism (*N* = 26), adrenal insufficiency (*N* = 41), hypophysitis/hypopituitarism (*N* = 151) mainly occurred within 12 weeks (100%, 92.3%, 80.8%, 63.4%, 71.5%, respectively), with median onset time ranging from 9 to 10 weeks. There was no significant difference in the onset time between these five ADRs (*P* = 0.570). (2) When using nivolumab, the onset time of endocrine ADRs was statistically different (*P* < 0.001). Hyperthyroidism occurred soon, with median time of 5 weeks (IQR 3–9). However, the onset time of other endocrine ADRs diffusely ranged from 0 to 48 weeks, with median onset time of 17 weeks for ICI-DM, adrenal insufficiency, hypophysitis/hypopituitarism, and 14 weeks for hypothyroidism. Thirty-one cases (15.7%, total 197) of ICI-DM even occurred more than 48 weeks after nivolumab initiation. (3) When using pembrolizumab, onset time of endocrine ADRs was roughly similar to that of nivolumab. Hyperthyroidism was also occurred soon after pembrolizumab initiation, with median time of 6 weeks (IQR 3–10). Differently, onset time of ICI-DM, adrenal insufficiency and hypothyroidism were slightly earlier than that of nivolumab, mainly within 24 weeks, with median onset time ranging from 12 to 14 weeks, but only ICI-DM and adrenal insufficiency had statistical significance (*P* = 0.034 and 0.047, respectively). (4) When using combination therapy (ipilimumab plus anti-PD-1/PD-L1), all endocrine ADRs we focused on mainly occurred within 48 weeks, with extremely low proportion of more than 48 weeks (all <3% in each ADR). Compared with nivolumab and pembrolizumab, respectively, onset time of hypothyroidism induced by combination therapy was significantly earlier (14 weeks (IQR 8–22) for nivolumab, 13 weeks (IQR 6–20) for pembrolizumab, 7 weeks (IQR 3–12) for combination therapy, both *P* < 0.001), while there was no significantly difference in ICI-DM, adrenal insufficiency, hypophysitis/hypopituitarism, and hyperthyroidism (*P* > 0.05, respectively). When comparing combination therapy with ipilimumab monotherapy, we found no difference in onset time of the analyzed endocrine ADRs, except for a slight statistically difference in adrenal insufficiency (13 weeks (IQR 8–24) for combination therapy, 10 weeks (IQR 6–14) for ipilimumab, *P* = 0.044).

## Discussion

To our knowledge, this is the largest and most extensive analysis of endocrine ADRs associated with ICIs collected from a worldwide pharmacovigilance database. Our study identified common and even rare endocrine ADRs and showed differences in endocrine toxicity spectra and onset time between different ICI therapies. Overall, four main findings emerged in our study.

First, based on VigiBase, ICI-related endocrinopathies had been increasing since 2017, with a male to female ratio of 1.5:1 and resulting in low incidence of fatal consequences.

The reports of ICI-related endocrinopathies had been increasing significantly over the past 3 years, with the largest contribution from anti-PD-1 monotherapy, followed by combination therapies (ipilimumab plus anti-PD-1/PD-L1). This may be due to a variety of reasons, including the rapid expansion of therapeutic indications for anti-PD-1 in different tumors over the past 5 years, high expectations for this emerging immunotherapy when encountering bottlenecks in anti-cancer process, and a higher rate of overall response in patients contributed by combination therapy [14–16]. In addition, we observed that among all analyzed ICI-related endocrinopathies, male accounted for a larger proportion than female. This seems to be inconsistent with the incidence of autoimmune disease in women. But it reported that melanoma and non-small cell lung cancer, top two common causes patients received ICI therapies, had a higher incidence in male than female [17, 18]. Whether the total cardinality of male cancer patients is the main factor of gender differences for ICI-related endocrinopathies remains to be further studied.

The severity of immune-related endocrine events is usually grades 1–2, and the incidence of adverse events in grades 3–4 does not exceed 2% [7]. Our study observed that only 64 cases (1.8%) of all ICI-related endocrinopathies resulted in fatal consequences, which were mainly related to hypophysitis, acute adrenocortical insufficiency hypothyroidism, diabetic ketoacidosis, fulminant type 1 diabetes mellitus, thyrotoxic crisis. We also found that nearly 70% of patients were recovered or attained remission from ICI-related endocrinopathies after standard treatment or a withdrawal of ICI. Some reports indicated that patients with irAEs have a higher rate of tumor response and a longer progression-free survival than those without irAEs [19, 20]. Consequently, given the safety and efficacy of ICI drugs, there is usually no need to reduce or discontinue drugs in cases of mild and moderate endocrine ADRs under vigilant clinical monitoring [5, 21].

Second, ICI-related endocrinopathies invaded almost all glands of the endocrine system, including pituitary (anterior and posterior lobes), adrenal, thyroid, parathyroid, gonad, pancreas. Some of these endocrine ADRs were common and some were rare.

We evaluated the signal strength of each endocrine ADRs in the study. All ICI-related endocrinopathies were observed compared with the whole database. ICI-related endocrinopathies invaded almost all glands of the endocrine system and our previous study showed that different endocrinopathies could occur simultaneously [22]. We reported the largest number of fulminant type 1 diabetes mellitus to date, which was mainly reported in individual cases [23, 24]. Fulminant type 1 diabetes had significant association with ICIs, especially anti-PD-1 monotherapy. Other meaningful endocrine ADRs were also observed, such as hypophysitis (or resulting in hypopituitarism), adrenal insufficiency, thyroid dysfunction (presenting as thyroiditis, hyperthyroidism or hypothyroidism), type 1 diabetes mellitus, diabetic ketoacidosis. Our study showed that insulin-deficient diabetes mellitus has gradually increased and is no longer an infrequent ICI-related endocrinopathy, which is different from prior literature reports [5, 6]. In addition, we also found some rare or critical ICI-related endocrinopathies, such as diabetes insipidus, hypoparathyroidism, thyrotoxic crisis, hypogonadism, which should be taken seriously by clinical oncologists and endocrinologists. Diabetes insipidus was extremely rare in ICI-related hypophysitis and had been reported in a few cases [25, 26]. In our study, 16 cases of diabetes insipidus were observed. Although diabetes insipidus was significantly associated only with ipilimumab, it was also observed in several reports induced by nivolumab or pembrolizumab. Endocrine toxicity invading the parathyroid gland has been rarely reported [27, 28] and no clear pathomechanism has been proposed. Eleven cases of hypoparathyroidism reported in the VigiBase database were induced by anti-PD-1 monotherapy (six cases) and combination therapy (five cases), and no reports of ipilimumab monotherapy. From this, hypoparathyroidism is presumably more related to anti-PD-1, which remains to explore in future clinical trials. Thyrotoxic crisis is a syndrome with acute exacerbation of thyrotoxicosis and was observed to be closely related to combination therapy. All 11 cases of thyrotoxic crisis were malignant melanoma patients and more clinical characteristics had been found in our previous study [29]. Hypogonadism was associated with ipilimumab, but there were only a few cases (*N* = 7). This may be due to transience of central hypogonadism [5] and high under-reporting because of patient privacy and low severity of disease itself.

Third, endocrine toxicity spectra induced by different ICI therapies were different. (a) Anti-CTLA-4 monotherapy was more related to hypophysitis (or resulting in hypopituitarism) and adrenal insufficiency. (b) Anti-PD-1 monotherapy was more related to thyroid dysfunction, T1DM, and fulminant T1DM. (c) Anti-PD-L1 monotherapy was more related to thyroid dysfunction, with the narrowest endocrine toxicity spectrum. (d) Combination therapy covered almost all endocrine ADRs and had stronger association with these ADRs than monotherapy.

Prior studies on the comparison of endocrine toxicity spectrum between anti-CLTA-4 and anti-PD-1 showed that hypophysitis is closely related to anti-CTLA-4 monotherapy, and thyroid dysfunction is closely related to anti-PD-1 monotherapy [4, 30]. Evidence from our study also supported this conclusion. Boutros et al. pointed out that PD‑1 inhibits T‑cell activity, mainly within the peripheral tissues and in the tumor microenvironment, which might explain the distinctive spectrum of anti‑PD‑1 differed from ipilimumab [31]. Compared with ipilimumab, insulin-dependent diabetes mellitus was also more related to anti-PD-1 monotherapy, which was reported to occur in 0.2–1.0% [32, 33] and is being reported more frequently as anti-PD-1/PD-L1 become more widely used. In view of this, clinicians who manage patients receiving anti-PD-1/PD-L1 treatment should monitor their blood glucose levels more carefully. Our study observed multiple forms of insulin-dependent diabetes mellitus, such as type 1 diabetes mellitus, fulminant type 1 diabetes mellitus, diabetic ketoacidosis, and hyperglycaemic hyperosmolar state. Case report also indicates that ipilimumab might induce type 1 diabetes mellitus or fulminant type 1 diabetes mellitus [34, 35], and eight cases of T1DM, one case of fulminant T1DM were reported in our study, respectively. Fulminant T1DM is the subtype of T1DM earliest described in Japan [36] and was shown to be closely related to anti-PD-1 in our study (88 cases), with no reports related to anti-PD-L1.

Actually, endocrine toxicity of anti-PD-L1 is difficult to compare sufficiently with anti-PD-1 drugs. Anti-PD-L1 are used less frequently than anti-PD-1 because these drugs were later approved. In addition, they differ in indications and anti-tumor types approved by the FDA. However, Martins et al. [37] proposed the hypothesis that since PD-L2 retains normal immune homeostasis, anti-PD-L1 antibodies may theoretically be less toxic. Our study discovered that anti-PD-L1 monotherapy was more closely related to thyroid dysfunction and can induce a small number of other endocrine ADRs, such as type 1 diabetes mellitus, hypophysitis and adrenal insufficiency. Moreover, the incidence differences of endocrine adverse reactions induced by anti-PD-L1 and anti-PD-1 may be due to different ligand-receptor interactions: PD-L1 antibodies binds CD80 as well as PD-1 receptors on activated T cells [30].

Combination therapy provides greater efficacy in many cancer immunotherapy [14], and ipilimumab with nivolumab is the first approved ICI combination therapy. However, an increase in efficacy appears to result in higher frequency and more severe ADRs. Our study confirmed that combination therapy seemed to increase the risk of all ICI-related endocrinopathies, which is consistent with prior studies [33, 38]. Notably, some clinically critical endocrine crises were observed to be more risky in combination therapy such as diabetes ketoacidosis (ROR: 3.1, 95% CI: 2.1–4.7), thyrotoxic crisis (ROR: 11.5, 95% CI: 2.4–53.8), acute adrenocortical insufficiency (ROR: 2.2, 95% CI: 0.9–5.7). In fact, not only the endocrine system, combination therapy also involves higher toxicity of some other systems, including cardiovascular toxicity (e.g., myocarditis) [8, 39], neurologic toxicity (e.g., noninfectious encephalitis/myelitis, Guillain–Barre syndrome, noninfectious meningitis) [40], skin toxicity [41] and digestive toxicity (e.g., colitis) [42]. Therefore, given the higher toxicity risk of combination therapy, clinicians should fully recognize it.

Fourth, onset time of major endocrine ADRs (ICI-DM, adrenal insufficiency, hypophysitis/hypopituitarism, and thyroid dysfunction) differed between different ICI therapies, typically within 12 weeks in anti-CTLA-4 monotherapy but diffusely ranging from 0 to 48 weeks in anti-PD-1 monotherapy. The onset time was shorter in combination therapy than anti-PD-1 monotherapy.

Our study analyzed in detail the onset time of major endocrine ADRs after the initiation of different ICIs. Prior studies have reported that the mean onset time of ICI-related endocrinopathies ranges from 4 to 36 weeks [5, 43], while ICI combination therapy has shorter onset time [38]. The severity grade of adverse events induced by anti-CTLA-4 monotherapy is higher than that of anti-PD-1 monotherapy [44], and anti-CTLA-4 appears to attack more rapidly and earlier. Our research demonstrated that ICI-DM, adrenal insufficiency, hypophysitis/hypopituitarism, hyperthyroidism, and hypothyroidism typically occurred within 12 weeks after ipilimumab initiation, with median onset time of 9–10 weeks. However, unlike ipilimumab, anti-PD-1 monotherapy had a much more dispersive range of onset time of endocrine ADRs mentioned above, except for hyperthyroidism. Moreover, ICI-DM may occur more than 48 weeks after anti-PD-1 initiation, up to 2 years according to our study. Therefore, it is difficult to predict the onset time of ICI-DM.

In addition, combination therapy may accelerate the occurrence of endocrine adverse events, with extremely high incidence within 48 weeks, and the median onset time was closer to that of ipilimumab monotherapy. Patients receiving combination therapy were observed to have a shorter median onset time of ICI-DM than anti-PD-1 monotherapy, but without statistical significance. Data from case report [45] described a woman with malignant melanoma rapidly developed into fulminant type 1 diabetes mellitus 3 weeks after receiving combination therapy. Importantly, our study observed that patients treated with combination therapy had significantly shorter onset time of hypothyroidism (7 weeks [IQR: 3–13]) than anti-PD-1 monotherapy (14 weeks [IQR: 8–22] for nivolumab, 12 weeks [IQR:6–20] for pembrolizumab; both *P* < 0.001).

We acknowledge the limitations of VigiBase database. Under-reporting of ADRs and lack of complete clinical information (e.g., laboratory tests, cancer severity and duration) are the common limitations of pharmacovigilance studies. In addition, two points need to specially take attention and understand when conducting such studies. (a) The IC value does not indicate a causal relationship between target drug and suspected ADR it caused but rather indicates the quantitative dependence of suspected ADR and target drug. (b) The ROR value does not indicate the real risk in clinical practice, but only indicates an increased risk of ADR reporting. Notwithstanding limitations for VigiBase, but pharmacovigilance assessment can provide momentous opportunity to monitor drug safety and identify new rare signals. We use this method to confirm that endocrine toxicity with ICIs could invade many organs or tissues of endocrine system, with different reporting frequencies between anti-CTLA-4 monotherapy and anti-PD-1/PD-L1 monotherapy. Moreover, some rare endocrine AEs were observed, including diabetes insipidus, hypoparathyroidism, thyrotoxic crisis, hypogonadism.

In general, ICIs-related endocrinopathies have been increasing based on real-world study of VigiBase. This study comprehensively evaluated and emphasized the differences in toxicity spectrum and onset time of endocrine ADRs among different ICI therapies, which should clinical oncologists and endocrinologists be aware of.

## Supplementary information

Supplementary Materials
